# Radix Entomolaris: Case Report with Clinical Implication

**DOI:** 10.5005/jp-journals-10005-1572

**Published:** 2018

**Authors:** Anshul Arora, Ashtha Arya, Latika Chauhan, Gaurav Thapak

**Affiliations:** 1-4 Department of Conservative Dentistry and Endodontics, Faculty of Dental Sciences, SGT University, Gurugram, Haryana, India

**Keywords:** Anatomical variation, Endodontic treatment, Mandibular molar, Radix entomolaris

## Abstract

Usually first mandibular molars have one mesial and distal root but in some cases there are anatomical variations wherein the number of roots and root canals vary. Presence of an additional lingual root distally in mandibular molars is called radix entomolaris (RE). Appropriate diagnosis is must before starting with root canal procedure in these teeth to ensure successful treatment outcome. The report describes the endodontic management of mandibular molar with RE.

**How to cite this article:**

Arora A, Arya A, Chauhan L, Thapak G. Radix Entomolaris: Case Report with Clinical Implication. Int J Clin Pediatr Dent, 2018;11(6):536-538

## INTRODUCTION

The main aim of the endodontic procedure is through the elimination of microbes from the root canal system and prevention of further reinfection, which is achieved by biomechanical cleaning of the pulp space followed by hermetic sealing with obturating material.

An awareness and comprehensive knowledge of the unusual root canal morphology can contribute to the success of the endodontic procedure. The majority of the mandibular first molars have one mesial and one distal root with two mesial canals and one distal canal.^1,2^

In most cases, the mesial root has two root canals, which may end in two separate apical foramina or they may merge to form a single foramen at the root end. The distal root generally has one bean shaped root canal. But mandibular molars with a varied number of roots and root canals have been observed during dental procedures, though incidences are rare. Carabelli was the first one to mention the presence of an additional root in mandibular first molar and called it as radix entomolaris (RE).^3^

This additional third root is commonly found distolingual. Radix entomolaris can be found in the first, second, and third mandibular molars, occurring least frequently in the second molar. When the extra root is present on the mesiobuccal side, it is called as radix paramolaris. Literature suggests the presence of RE in less than 5% population in white Caucasian, African, Eurasian and Indians whereas it is present with a frequency of 5–30% in races with Mongoloid traits such as the Chinese, Eskimos, and Native Americans.^4–7^ This article highlights the clinical approach for identification and modifications in endodontic management of mandibular first molar with RE.

## CASE REPORT 1

A 27-year-old male patient reported to the Department of Conservative Dentistry and Endodontics, SGT Dental College, with a chief complaint of severe pain in the right lower back tooth region since last three days. The pain was intermittent in nature and aggravated on taking hot food and beverages, and lasted for 2–3 hours.

**Fig. 1:**
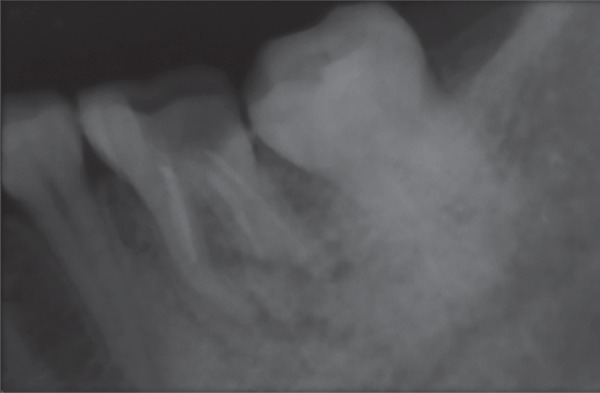
Preoperative

On clinical evaluation, it was seen that there was secondary caries associated with restored right mandibular first molar. A diagnostic radiograph of mandibular first molar showed restoration close to pulp and presence of an additional root (Fig. 1). Another radiograph was taken at 300 mesial and distal angulation to confirm the same. Access cavity preparation was done under local anesthesia with an endo access bur (Dentsply, Switzerland). The first distal canal was located towards the buccal side indicating the presence of one additional canal on the lingual side.

The shape of the access cavity was modified from triangular to a trapezoidal form to locate the fourth canal. DG-16 endodontic explorer was used to locate the root canal orifices and 15 # K-file (Mani, Japan) was used to establish patency of the canals. Working length was determined using apex locator (Root ZX, J. Morita) and reconfirmed radiographically. Biomechanical preparation was done with rotary ProTaper Next (Dentsply, Switzerland) file system. During instrumentation, 1.3% sodium hypochlorite was used as an irrigant and 17% EDTA was used as final flush.

**Fig. 2:**
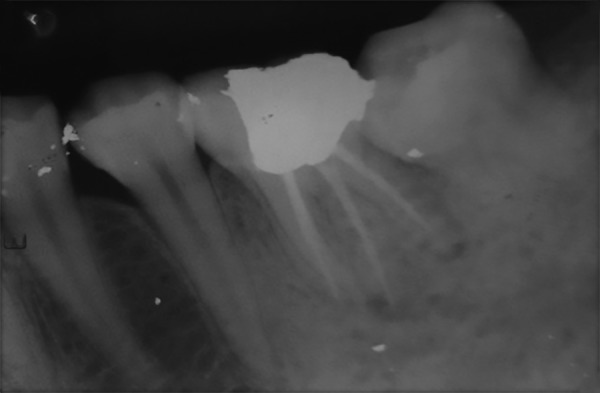
Postoperative

Obturation was performed with gutta-percha points using cold lateral condensation technique (Fig. 2). Restoration of access cavity was done with composite resin (tetric-N-ceram, ivoclar vivadent) and a post-obturation radiograph was taken. At 6-month follow-up, the patient was asymptomatic and radiographic evaluation showed no evidence of pathology.

## CASE REPORT 2

**Fig. 3:**
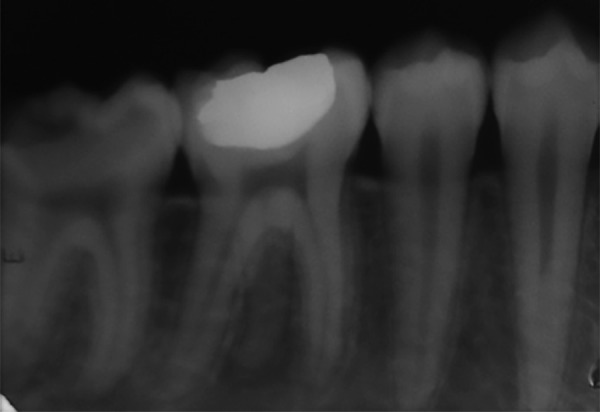
Preoperative

A 22-year-old female patient reported to our Department with a history of pain in the lower right back tooth for 1 month. On clinical examination, right mandibular first molar was found to be carious and the diagnosis was established as irreversible pulpitis. Radiographic interpretation revealed the presence of the third root but not associated with any periapical changes (Fig. 3).

Root canal procedure of the tooth was planned and patient consent obtained. Local anesthesia was administered and after access cavity preparation canal orifices were negotiated with DG-16 explorer. The fourth distolingual canal orifice was negotiated more lingually, away from the rest of the three orifices. The canal lengths were determined radiographically with #10 K file, and cleaning and shaping of the root canal system were done with rotary ProTaper file system with apical preparation till F2. 1.3% sodium hypochlorite was used for disinfection of the pulp space and 17% EDTA was used as a final irrigant. After biomechanical preparation, temporary restoration was done and the patient was recalled after 4 days.

**Fig. 4:**
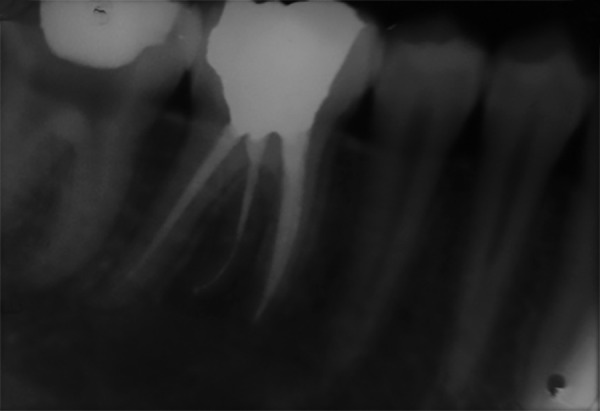
Postoperative

At subsequent appointment patient was asymptomatic. Master cone radiograph was taken, canals were dried with absorbent paper points, and obturation was done with gutta-percha cones using AH Plus sealer (Dentsply De Trey, Germany) (Fig. 4).

## DISCUSSION

Clinical triad of diagnosis, adequate chemomechanical preparation, and three-dimensional obturation determine the success of root canal therapy.

The first stage of endodontic triad, i.e., correct diagnosis is one of the most important steps towards the success of the endodontic procedure. One of the main reasons for the failure of root canal treatment is negligence in removing pulpal tissue and microbes from all the pulp canals. Hence, appropriate radiographic diagnosis play a crucial role in the successful outcome of endodontic therapy.^8^

So, radiographs were taken at different angulations to minimize the chances of “missed canals”.^9^ Radix entomolaris has a prevalence rate of less than 5% in the Indian population and such cases are not commonly observed during dental treatment. The exact etiology of radix entomolaris is still not known but according to some authors it may be due to disturbance during odontogenesis or may be due to the high degree of genetic penetrance.^10^

To avoid any iatrogenic mistake, minimum of two angulated diagnostic radiographs are a must along with the careful clinical examination. If RE is diagnosed before commencing the endodontic treatment, a modified trapezoidal access cavity can be prepared and additional canal orifice can be searched for.

With a good understanding of the law of symmetry, various methods like visualizing the dentinal map and canal bleeding points using magnification, ultrasonic tips, staining the chamber floor with 1% methylene blue dye, performing champagne bubble test, and cone beam computed tomography imaging will help identify the missed canal.

De Moor et al.^9^ studied the morphology of radix entomolaris and concluded that in the majority of cases, these canals were curved. Hence after initial root canal exploration with small files (size 10 or less) together with radiographic working length and curvature determination, the creation of straight-line access and preparation of glide path has to be emphasized to avoid procedural errors.

## CONCLUSION

The complexity of the root canal system often pose a challenge to the clinician. Failure to identify the RE can affect the prognosis of endodontic treatment. Preoperative radiographs at 300 mesial and distal angulations and their proper interpretation are mandatory to prevent any lapse in the diagnosis of RE. Thus, an accurate diagnosis and thorough knowledge and about the variation in root canal morphology, prevalence and canal configuration of radix entomolaris is prerequisite for endodontic success.
